# Qualitative thematic analysis of consent forms used in cancer genome sequencing

**DOI:** 10.1186/1472-6939-12-14

**Published:** 2011-07-19

**Authors:** Clarissa Allen, William D Foulkes

**Affiliations:** 1 Program in Cancer Genetics, Departments of Oncology and Human Genetics, McGill University, Montreal, Quebec, Canada; 2 Biomedical Ethics Unit, McGill University, Montreal, Quebec, Canada

## Abstract

**Background:**

Large-scale whole genome sequencing (WGS) studies promise to revolutionize cancer research by identifying targets for therapy and by discovering molecular biomarkers to aid early diagnosis, to better determine prognosis and to improve treatment response prediction. Such projects raise a number of ethical, legal, and social (ELS) issues that should be considered. In this study, we set out to discover how these issues are being handled across different jurisdictions.

**Methods:**

We examined informed consent (IC) forms from 30 cancer genome sequencing studies to assess (1) stated purpose of sample collection, (2) scope of consent requested, (3) data sharing protocols (4) privacy protection measures, (5) described risks of participation, (6) subject re-contacting, and (7) protocol for withdrawal.

**Results:**

There is a high degree of similarity in how cancer researchers engaged in WGS are protecting participant privacy. We observed a strong trend towards both using samples for additional, unspecified research and sharing data with other investigators. IC forms were varied in terms of how they discussed re-contacting participants, returning results and facilitating participant withdrawal. Contrary to expectation, there were no consistent trends that emerged over the eight year period from which forms were collected.

**Conclusion:**

Examining IC forms from WGS studies elucidates how investigators are handling ELS challenges posed by this research. This information is important for ensuring that while the public benefits of research are maximized, the rights of participants are also being appropriately respected.

## Background

The past decade has been characterized by a breathtaking acceleration in genome sequencing technology [1]. While at the beginning of the decade it took years to sequence the first complete human genome, over 200 human genomes have been sequenced in the past year alone, and experts predicts that approximately 25,000 will be sequenced by the end of 2011 [2]. One of the most exciting applications of this new technology is deciphering the genetic basis of complex diseases [3]. Cancer in particular provides an ideal and noteworthy focus for this approach. As a highly heterogeneous genetic disease mainly driven by somatic mutations that can occur at a multitude of locations, cancer is particularly amenable to this type of study. By enabling researchers to compare the host genome to that of the cancer itself, whole genome sequencing (WGS) technology promises to not only identify predictors of, and targets for, therapy, but also discover novel molecular biomarkers to aid in the early detection of cancer. In combination, these will likely positively influence prognosis [4]. Consequently, a number of large-scale research studies using WGS technology to identify genetic components of cancer have recently been initiated. Projects such as The Cancer Genome Atlas and the International Cancer Genome Consortium are obtaining biological samples from thousands of subjects around the world in an attempt to catalogue the myriad genetic and genomic alterations that are critical to carcinogenesis. This research raises a number of ethical, legal, and social (ELS) issues that need to be addressed.

Investigators engaged in large-scale WGS research projects must consider how to manage issues such as sample and data sharing, protecting participant privacy and confidentiality, communicating the risks and benefits of research to participants, returning individual results, and enabling participant withdrawal from research when requested. These issues are common to the majority of human subject research, but the unprecedented volume of genomic data that is being collected through WGS, the inherent unpredictability of what this technology will reveal in comparison to targeted sequencing and the rapid rate at which our interpretive abilities are advancing obliges us to re-examine these issues in the context of this research. Our goal in this study therefore was to perform a qualitative analysis of consent forms in order to discern how investigators currently engaged in cancer genome sequencing across a variety of jurisdictions are addressing these issues. Focusing on cancer genome research enables comparison across studies, allowing us to add to knowledge of current practices, identify similarities and differences, and determine whether common practices are emerging. As informed consent (IC) documents are the main vehicle through which information about ELS issues is communicated to research participants, we chose to focus our study on the analysis of these forms. We anticipated that trends would emerge both across jurisdictions and over time.

## Methods

We obtained contact details for researchers engaged in cancer genome sequencing by visiting the websites of large-scale WGS projects, found by doing internet searches for 'whole genome sequencing' and navigating the website of the National Institute of Health. We also searched http://Pubmed.gov for papers reporting studies that involved sequencing whole cancer genomes, using the key words 'whole genome sequencing' and 'next-generation sequencing' and limiting the searches to humans only, within the past five years, under the subset 'cancer'. Additional file 1, Table 1 presents a list of these studies that is current at the time of publication, with a succinct summary of their key findings. Our personal knowledge of ongoing research projects provided further guidance in this search.

**Table 1:** Summary of Results

Form	Source	Research scope	Type of results returned	Re-contacting choice?	Purpose of re-contact	Data sharing with?	Holder of coded data key	Risk of dis-crimination discussed?	Scope of consent	Right to withdrawal
2004	UK	Genetic basis of cancer	General only	No comment	No comment	Affiliated research	PI	No	Study only	Consequences unclear

2005	US	A particular system	General only	No comment	No comment	Affiliated research	PI	Yes	Related Research	Samples destroyed but not info that has been used

2005	Australia	General scientific research	Individual, no option	Yes, may re-contact	Follow-up info	Any who apply	Third party	No	Choice Provided	Samples destroyed but not info that has been used

2006	US	Genetic basis of cancer	No comment	No, will re-contact	Follow up info and future research	Any who apply	PI	Yes	Blanket consent	Samples destroyed but not info that has been used

2008	UK	A particular system	None	No comment	No comment	Any who apply	Third party	No	Blanket consent	All info removed/destroyed

2008	US	Genetic basis of cancer	General only	No, will re-contact	Follow up info	Any who apply	PI	Yes	Blanket consent	Samples destroyed but not info that has been used

2008	US	General scientific research	General only	No comment	No comment	Any who apply	Identifying info not collected	Yes	Blanket consent	No withdrawal (samples anonymized)

2008	Canada	Genetic basis of cancer	None	No, will re-contact	Follow up info	No comment	Third party	Yes	Related research	Consequences unclear

2009	Canada	Genetic basis of cancer	Individual, with option	No comment	No comment	Affiliated researchers	PI	Yes	Study only	All info removed/destroyed

2009	Canada	Genetic basis of cancer	No comment	Yes, may re-contact	Future research	Affiliated researchers	Third party	Yes	Related research	Consequences unclear

2009	Belgium	Genetic basis of cancer	Individual, with option	No comment	No comment	Affiliated researchers	PI	No	Blanket consent	Samples destroyed but not info that has been used

2009	US	Genetic basis of cancer	Individual, with option	Yes, may re-contact	Follow up info	Any who apply	Identifying info may be shared	Yes	Choice provided	Samples destroyed but not info that has been used

2009	US	Genetic basis of cancer	Individual and general, no option	Yes, may re-contact	Follow up info and future research	Any who Apply	PI	Yes	Blanket consent	Samples destroyed but not info that has been used

2010	US	Cancer research	None	Yes, may re-contact	Follow up info and future research	No comment	Third party	Yes	Choice provided	No comment

2010	US	Un-determined	General only	No, will re-contact	Follow up info	Any who apply	Third party	Yes	Blanket consent	Samples destroyed but not info that has been used

2010	US	Genetic basis of cancer	No comment	Yes, may re-contact	Follow up info	Affiliated researchers	Third party	No	Related research	Samples destroyed but not info that has been used

2010	US	Genetic basis of cancer	None	Yes, may re-contact	Follow up info	Any who apply	PI	Yes	Blanket consent	Samples destroyed but not info that has been used

2010	Netherlands	General scientific research	None	No comment	No comment	No comment	Third party	No	Blanket consent	No comment

2010	UK	Undetermined	General only	No comment	No comment	Any who apply	Third party	Un-determined	Blanket consent	Samples destroyed but not info that has been used

2010	Australia	Genetic basis of cancer	Individual and general, with option	Yes, may re-contact	Follow up info	Affiliated researchers	Third party	No	Related research	All info removed/destroyed

2011	US	A particular system	None	Yes, may re-contact	Follow up info and future research	Any who apply	Third party	Yes	Choice provided	Samples destroyed but not info that has been used

2011	US	Cancer research	General only	No, will re-contact	Follow up info	Affiliated researchers	Identifying info may be shared	Yes	Blanket consent	Samples destroyed but not info that has been used

2011	US	A particular system	Individual, no option	No, will re-contact	Individual results	Any who apply	Third party	Yes	Choice provided	Samples destroyed but not info that has been used

2011	US	General scientific research	General only	No comment	No comment	Affiliated researchers	Third party	Yes	Blanket consent	Samples destroyed but not info that has been used

None	Australia	Genetic basis of cancer	Individual, no option	No, will re-contact	Individual results	Any who apply	PI	No	Blanket consent	Samples destroyed but not info that has been used

None	US	Genetic basis of cancer	None	No, will re-contact	Follow up info	Any who apply	PI	Yes	Blanket consent	Samples destroyed but not info that has been used

None	US	Genetic basis of cancer	Individual, with option	No, will re-contact	Future research and individual results	Affiliated researchers	PI	Yes	Blanket consent	Consequences unclear

None	US	General scientific research	None	No comment	No comment	Affiliated researchers	Third party	Yes	Choice provided	Samples destroyed but not info that has been used

None	US	Genetic research	Individual, no option	No, will re-contact	Follow up info and future research	Any who apply	PI	Yes	Blanket consent	Samples destroyed but not info that has been used

None	US	Genetic basis of cancer	None	No, will re-contact	Follow up info	Any who apply	PI	Yes	Blanket consent	Samples destroyed but not info that has been used

We contacted researchers via email and/or phone. Individuals were selected on the basis of either being listed as a primary contact at tissue source sites for large-scale WGS projects, or as corresponding authors for journal articles. We explained the details of our research project, and requested that the researchers send us templates of the informed consent documents used by their institutions or research groups when obtaining tissue samples for cancer genome sequencing research. It was explained to potential participants that neither specific institutions nor individuals would be identified in our results or discussion. In total 54 researchers were identified. Of these, 12 were excluded on the basis of being part of the same research groups as other researchers on the list. Of the 42 remaining researchers, 30 sent informed consent documents, giving us a response rate of 71.4%.

We performed a qualitative thematic analysis on the IC forms that were received. We assigned each document a value under the following headings: (1) stated purpose of sample collection, (2) scope of consent requested, (3) data sharing protocols (4) privacy protection measures, (5) described risks of participation, (6) subject re-contacting, and (7) protocol for withdrawal. Values were specific to each heading, and represented what was stated in the informed consent document in relation to the topic at hand.

## Results

For the 30 informed consent documents obtained, the country-of-origin breakdown was as follows: one from Belgium, one from the Netherlands, three from Australia, three from Canada, three from the UK and nineteen from the US. The most recently dated form was from May 2011, while the oldest was dated March 2004. Six of the forms were not dated, while three others were from 2011, six were from 2010, five were from 2009, five were from 2008, one was from 2006, and two were from 2005. The key findings from our thematic analysis, in summary form, are presented in Table 1.

We divided the IC forms into three categories based on the described purpose of tissue sample acquisition. Of the 30 documents obtained, the majority (n = 17) stated that samples were being collected for the purpose of cancer genetics research. Six were seeking samples for the purpose of broader medical research on a particular system or tissue type. Five were seeking blanket consent for any type of generalized medical research. Two forms were templates, in which the purpose of research was to be filled in upon use.

In addition to the stated purpose of the sample collection, we identified the scope of the consent that was being requested. Only two of the documents were seeking consent exclusively for the specific study described. Six were seeking consent to use the samples collected for research related to the study at hand, for example on the same disease, and intended to keep the samples indefinitely for this purpose. Six documents provided a choice as to the scope of consent, while sixteen stated that once obtained, samples might be used for any type of research at any future date.

The documents discussed not only sharing of samples with other researchers, but the sharing of data as well. Eleven of the documents stated that data might be shared with other researchers who were in some way affiliated with the project being described, for example in terms of working with the same organization or on the same disease. Sixteen of the documents stated that data could be shared with anyone, though more sensitive data would be placed in secure databases to which researchers would need to apply in order to gain access. Three of the documents did not reference data sharing at all.

Although many of the documents asked for broad consent and stated fairly liberal data sharing intentions, all but one (which did not collect identifying information) of the documents stated that data would be coded, with personal identifiers removed, and only a single person or small group of people having access to keys for linking coded information to specific research subjects. Although in three cases it was not stated, in approximately half (n = 13) of the forms the primary investigator was identified as the person with key access, while the other half (n = 13) stated that only data bank personnel, not researchers, would be able to re-link personal identifiers to information generated from research. Despite these confidentiality measures, twenty-one of the documents, predominantly those from North America, stated the danger of information of research participation and even individual results becoming known, and noted potentially associated repercussions, such as discrimination, as a risk of providing samples. Nine documents did not specify such socio-economic factors as a risk of participation in research, while one left the 'risks' section blank, to be filled in at the time of use.

Nine of the documents provided potential subjects with the choice to be re-contacted. In five cases this was to obtain follow-up health and demographic information, in one case to solicit participation in future research studies, and in three cases for both follow-up and future studies. Eleven of the documents stated simply that participants would be re-contacted; six for follow-up information, two to return individual results, one for both follow-up information and future studies, and two for both future studies and to return individual research results. Ten of the documents did not refer to re-contacting research participants.

Ten of the documents collected stated that general study results would be made available to participants. Of those ten, one stated that individual results would also be returned, one provided participants with a choice in obtaining individual results, seven stated that no individual results would be returned, and one did not discuss individual results. Of the remaining twenty, four stated that individual results would be returned, four provided participants with a choice regarding the return of individual results, nine stated that individual results would not be returned, and three did not discuss this issue.

Finally, we looked at the documents' explanation of subjects' ability to withdraw from research participation. Two documents did not refer to the option to withdraw. Of the remaining twenty-eight, three stated that should subjects choose to withdraw, all samples would be destroyed and information removed from the research project. Twenty stated that upon withdrawal samples would be destroyed, but information that had already been incorporated into research would not be removed. One document stated that as no identifying information would be collected, withdrawal would not be possible. The remaining four documents described participants' right to withdraw from research, but were ambiguous about what effect this would have on samples and data.

## Discussion

Informed consent in a research context is an autonomous action by a subject that authorizes a professional to involve that subject in a particular research endeavor [5]. To facilitate this authorization, IC forms must describe the purpose and scope of the study, potential risks and benefits of participation, how issues of privacy and confidentiality will be addressed, whether samples and/or data will be available to other researchers and whether research results will be available to participants and/or the public [6]. General information on informed consent can be found in guidance documents such as the United Nations' Ethical, Social, and Cultural Organizations' "Universal Declaration on the Human Genome and Human Rights" (1997) and the Council for International Organization of Medical Sciences' "Ethics and Research on Human Subjects: International Guidelines" (1992). Examining how these issues are addressed in IC forms that are being used in current cancer genome sequencing research however provides a more up-to-date and informative illustration of how investigators in this domain are balancing their ethical obligations to research subjects with the ultimate goal of research, which is to produce generalizable scientific knowledge for the benefit of society at large [7].

### Obtaining Informed Consent for Future Research and Data Sharing

The goal of large-scale genome sequencing projects is to create databases of genomic and phenotypic information to be widely disseminated in support of research advances [8]. Maximizing the utility of these databases by making as much information available to as many researchers as possible is undoubtedly beneficial to the advancement of science and so to the public good. This is especially true when databases are linked to identifiable and regularly updated personal information, such as medical records, so that correlations between genetic factors, treatment strategies and health outcomes can be made [6]. Broad consent from participants enables the future application of data to novel contexts that are not foreseeable at the time of collection [9]. The IC forms collected for this study illustrate a trend towards the broad sharing of samples and data, as 73% of the forms articulated the intention to use the samples for future, possibly unrelated research and 90% expressed the intention to share their research data with other, possibly unaffiliated, researchers. However, this otherwise laudable strategy must be balanced with ethical and legal obligations to obtain adequately informed consent from individual participants [6].

The broad data sharing through publicly accessible databases that is occurring in the context of large scale sequencing studies presents a challenge to the traditional conception of informed consent. At the point of sample collection, neither the researchers who will request access to the data nor the research questions that will be asked can be fully predicted [10]. This is especially true considering the extended period of time for which samples are being maintained and the rapid rate at which technology is advancing. Consent that is broad enough to cover all of the potential, unknown future of uses of research data is arguably too broad to be meaningful [8]. As a result of this, there is a growing body of literature in biomedical journals calling for a re-conceptualization of the role of autonomy and informed consent in research in order to accommodate the needs of science. Proponents argue that these databases are a revolutionary platform for medical research, and can exist only by relying on models of open consent and public data access [9]. Obtaining broad consent to multiple purposes of research and future consent to as yet unspecified research, as the majority of the forms we collected did, is therefore being proposed as a legitimate mechanism for the advancement of science [11].

The Unites States federal research regulations, referred to as 45CFR46, do not consider research involving only coded private information or specimens to involve human subjects, and so does not require informed consent for such research (45CFR46.102(f)). Thirteen of the forms we examined stated that researchers would not have access to the codes linking data to individuals, and so research arising from data sharing in these cases falls into this category. That being said, there are a number of reasons to advocate for stronger subject protections in relation to genomic databases. Especially when genotypic data are linked to regularly updated phenotypic data, which many argue is an essential feature of these databases [6], there is a risk that even de-identified sequence data can be matched against third party database to effectively re-identify an individual, and so privacy and confidentiality cannot be guaranteed [12]. Additionally, as WGS data becomes amenable to the study of complex traits beyond disease, such as behaviour, there may be significant risk to both individual autonomy and cultural identity [8]. Most research ethics guidelines, including the Declaration of Helsinki, agree that the objectives of science should not supersede individual rights. As the control over information that implicates personal integrity is recognized as a fundamental human right [6], the role of open-access genomic databases in advancing medical research alone is insufficient to trump the ethical obligation to obtain truly informed consent. The tension between individual and social interests in this context therefore remains a hotly debated issue [13].

### Genetic Research and Socio-economic Risk

Ensuring that potential research participants are providing consent that is substantially informed requires that the foreseeable risks and benefits of research are fully disclosed. These include the risk that an individual's participation in WGS research and resulting genetic information could become known to unauthorized parties. While all of the documents we collected stated that personal identifiers would be removed from samples and data so as to protect individual privacy, 70% of the forms also warned potential subject that privacy and confidentiality measures were not infallible. As each individual's genomic code is unique, knowledge of even a small number of genetic variants can result in samples being matched to individuals with a relatively high level of confidence [14]. Even when research data are de-identified, the increasing linking and interoperability of various health information databases heightens the possibility that individuals may be re-identified [15]. The ease with which research data can be disseminated via the internet increases the possibility that such information may be obtained by entities not subject to privacy regulations [14]. It is therefore appropriate for researchers to warn potential participants that privacy and confidentiality cannot be guaranteed [9]. What is less clear, however, is what dangers the potential disclosure of individual genetic information presents.

There has been much concern regarding the potential misuse of genetic information in the bioethics literature, not to mention in popular culture. However, little evidence of such discrimination actually occurring has been documented [16]. That being said, genetic information does possess some traits that when considered collectively, imply that individuals ought to be wary of sharing their genetic information. Firstly, genomic data are immutable; other than a relatively small number of somatic mutations, an individual's DNA sequence does not markedly change over time. Given unforeseeable technological and interpretational advances, particularly in terms of understanding the genotypic-phenotypic relationship, this means that public disclosure may have long lasting and unanticipated effects. Secondly, genetic information is predictive. While it is ultimately the gene-environment interaction that will determine what genetic predispositions manifest, unjust discrimination may occur on the basis of such future possibilities [14]. Thirdly, genetic data can provide potentially sensitive information not only about an individual, but about genetically-related family members and thus familial relationships as well [17]. This arguably increases the burden on each individual to protect their genetic information: it is not only their own privacy but also that of their relatives that is at stake. The risk of genetic discrimination is likely small, especially as in many countries there are laws in place to protect individual privacy [18]. However, researchers ought to articulate the potential dangers associated with individual data entering the public domain when discussing the risks of participating in research with their potential subject.

### Re-contacting Research Subjects and Returning Individual Results

The forms we collected discussed re-contacting for three purposes; to suggest further research participation, to obtain further information about participants, and to return individual research results. While some commentators argue that re-contacting participants is unduly burdensome in terms of the time and resources it consumes [19], it can also provide valuable information. One of the greatest challenges that genetic research currently faces is elucidating the gene-environment interactions that contribute to health outcomes and so contacting participants to update this information may be invaluable to research [20,21]. Investigators must consider however that particularly in the context of cancer genome sequencing, participants may not wish to be re-contacted. All of the documents we examined requested samples of tissue that was being removed for treatment purposes regardless of participation in research. Tumor removal surgery can obviously be a stressful time for patients and their families and even those who agree to have their samples used in research may not want to be reminded of this period through re-contacting. Nine of the forms we analyzed provided participants with a choice as to whether they wanted to be re-contacted; researchers should consider providing such an option if they would like to re-contacting their participants.

Re-contacting participants in order to return individual results is an issue that has recently been highly debated in the literature. Some argue that individual results should only be returned if they have clear clinical utility, and as the contribution of genetics to disease is still fairly rudimentary, the danger of returning unsubstantiated or even inaccurate results outweighs the benefits of returning results that are believed to be valid [22]. Furthermore, returning individual results may promote a therapeutic misconception, where the subject believes that the primary aim of the research is to advance their own best interests, rather than generate generalizable knowledge [23]. Additionally, in order to disclose individual results responsibly, participants must have access to genetic counselors who can explain to them the implications of their results, as well as to necessary follow up treatment [24]. Commentators argue that these requirements place too great a burden on the research endeavor [25]. Finally, cancer genome sequencing poses a particular difficulty in this respect, as the disease is caused by somatic mutations that may occur in a multitude of locations. Researchers sequencing cancer genomes are generally not seeking germline susceptibility loci, as is the case in research on other genetic diseases. The wide breadth of investigation to identify the point of carcinogenesis however may well identify such susceptibility loci incidentally. The issue of how to manage such incidental results is controversial [26], but was not mentioned in any of the forms we collected.

Despite these arguments, in recent years there has been growing consensus that researchers have an ethical obligation to return individual research results [27]. Arguments based in such fundamental ethical principles as respecting participant autonomy, beneficence and reciprocity, in conjunction with the scientific advances that are making it increasingly feasible for research to produce analytically valid and clinically useful results, substantiate this claim [28]. In addition, providing participants of large-scale genomic studies with the option to receive individual results promises to improve public understanding of genetics [29], which in turn may help to reduce the risk of genetic discrimination. Researchers ought to consider these arguments when designing their research protocols. One third of the forms we collected provided participants with individual results or the option to obtain them, which indicates that doing so is a feasible endeavor in this context.

### Withdrawing from Research

Withdraw from research is generally considered to be a fundamental right [30,31]. People's perceptions change over time, and so this is especially important in long-term studies [10]. Of the documents we collected, only two did not discuss this right. An issue that arises here is the difficulty of removing an individual's samples and data from the research project if they have already been shared with other researchers and/or analyzed and incorporated into aggregate results. Twenty of the forms we collected acknowledged this by stating that should the subject chose to withdraw, their samples would be destroyed but data already in use would not be removed. In response to this problem anonymization has been suggested as an alternative to withdrawing, particularly so that samples and data can continue to be used in long-term studies. This is an imperfect solution however, as samples would no longer be useful for diagnostics, the characterization of group members would not be prevented, and samples could potentially still be re-identified [10]. A second suggestion is that in an attempt to preserve the usefulness of samples, researchers could provide participants with withdrawal options. For example participants could withdraw from further contact and linkage of their samples with health records, but continue to allow use of samples and data, as opposed to withdrawing permission for future use of samples all together [17]. More research and consideration is required to address this contentious issue, but in the meantime, researchers ought to make it clear to subjects that once samples have been analyzed and data disseminated for use by third parties, in all likelihood it will be impossible to effect a meaningful retraction at a later date [9].

## Conclusion

This study illustrates that there is a high degree of similarity in how cancer researchers engaged in WGS are protecting participant privacy and also there is a strong trend towards both using samples for additional, unspecified research and sharing data with other researchers. IC forms were more varied in terms of how they discussed re-contacting participants, returning results, and facilitating participant withdrawal from research. While this variability might arise as a result of differences in local ethics review board requirements, the research ethics literature makes a number of recommendations in respect to these issues, which if taken into consideration may facilitate a degree of standardization across studies.

Some limitations of this study include that restricting our analysis to the examination of IC documents means that some relevant ethical issues, such as for example participant recruitment, the selection of research questions, and potential conflicts of interest, could not be addressed.

Additionally, while WGS is a relatively new technology, we compared forms across a period of eight years. However, as no particular trends seem to emerge over time, our conclusion that the IC forms vary in a number of respects is clearly not a reflection of the time period chosen. It is also worth noting that while we focused on WGS that is occurring as a part of cancer research studies in order to facilitate comparison, the majority of the documents we analyzed were seeking consent for broader research. As a result of this, our results are highly generalizable to other studies involving WGS.

As WGS has the advantage of identifying effectively all genetic changes (i.e. mutation, rearrangement and copy number) in a genome, it is quickly becoming the 'gold standard' of genetic analysis [32]. However, while the technology is advancing at a rapid pace, challenges to its implementation (i.e. to "clinical-grade genomic sequencing") remain. WGS produces a huge amount of information; development of computational analysis and storage techniques through advances in bioinformatics is required in order to manage these data. Advances in our currently limited knowledge of the functional significance of many human genes, as well as in our understanding of gene-environment interactions, are also needed before the full value of WGS can be realized [33]. Finally, ELS issues associated both with WGS research and the implementation of WGS in clinical care must be addressed if this research is to proceed in such a way as to both maximize the social good, and simultaneously respect the rights of participants.

We hope that efforts to generate IC forms that can be generalizable across jurisdictions will be aided by thematic analyses such as the one conducted here.

## Competing interests

The authors declare that they have no competing interests.

## Authors' contributions

CA collected and analyzed informed consent documents, and drafted the manuscript. WDF revised the manuscript critically for important intellectual content. Both authors participated in the design of the study and read and approved the final manuscript.

## Pre-publication history

The pre-publication history for this paper can be accessed here:

http://www.biomedcentral.com/1472-6939/12/14/prepub

## Supplementary Material

Additional file 1 **Summary of published cancer genome sequencing projects**. Provides information on notable cancer genome sequencing projects that have been published. Click here for file

## References

[B1] Collins F Has the Revolution Arrived? Nature 2010 464 674 675 10.1038/464674a 20360716 PMC5101928

[B2] Drmanac R The Advent of Personal Genome Sequencing Genet Med 2010 13 188 190 10.1097/GIM.0b013e31820f16e6 21311341

[B3] Bonetta L Whole-Genome Sequencing Breaks the Cost Barrier Cell 2010 141 917 919 10.1016/j.cell.2010.05.034 20550926

[B4] Stratton MR Campbell PJ Futreal PA The cancer genome Nature 2009 458 719 724 10.1038/nature07943 19360079 PMC2821689

[B5] Faden RR Beauchamp TL A History and Theory of Informed Consent 1986 New York: Oxford University Press

[B6] Caulfield T Biobanks and blanket consent: the proper place of the public perception and public good rationales King's Law J 2007 18 209 22621776343

[B7] Meltzer LA Undesirable Implications of Disclosing Individual Genetic Results to Research Participants Am J Bioeth 2006 6 28 30 17085401 10.1080/15265160600935811

[B8] McGuire AL Caulfield T Cho MK Research Ethics and the Challenge of whole-genome sequencing Nat Rev Genet 2008 9 152 156 18087293 10.1038/nrg2302 PMC2225443

[B9] Lunshof JE Bobe J Aach J Angrist M Thakuria JV Vorhaus DB Hoehe MR Church GM Personal genomes in progress: from the Human Genome Project to the Personal Genome Project Dialogues Clin Neurosci 2010 12 34 44 10.31887/DCNS.2010.12.1/jlunshof PMC3181947 20373666

[B10] Secko DM Preto N Niemeyer S Burgess MM Informed consent in biobank research: A deliberative approach to the debate Social Science & Medicine 2008 68 781 789 19095337 10.1016/j.socscimed.2008.11.020

[B11] Hansson M Dillner J Bartram CR Carlson J A Helgesson G Should donors be allowed to give broad consent to future biobank research? Lancet Oncology 2006 7 266 269 10.1016/S1470-2045(06)70618-0 16510336

[B12] Malin BA An evaluation of the current state of genomic data privacy protection technology and a roadmap for the future J Am Med Inform Assoc 2005 12 28 34 15492030 10.1197/jamia.M1603 PMC543823

[B13] Caulfield T Upshur R Daar A DNA databanks and consent: a suggested policy option involving an authorization model BMC Medical Ethics 2007 4 10.1186/1472-6939-4-1 PMC140033 12513704

[B14] McGuire AL Fisher R Cusenza P Hudson K Rothstein MA McGraw D Matteson S Glaser J Henley DE Confidentiality, privacy, and security of genetic and genomic test information in electronic health records: points to consider Genet Med 2008 10 495 499 10.1097/GIM.0b013e31817a8aaa 18580687

[B15] TCGA Human Subjects Protection and Data Access Policies 2011 http://cancergenome.nih.gov/abouttcga/policies/policiesguidelines

[B16] Barlow-Stewart K Taylor SD Treloar SA Stranger M Otlowski M Verification of consumers' experiences and perceptions of genetic discrimination and its impact on utilization of genetic testing Genet Med 2009 11 193 201 10.1097/GIM.0b013e318194ee75 19287242

[B17] Ries NM LeGrandeur J Caulfield T Handling ethical, legal and social issues in birth cohort studies involving genetic research: responses from studies in six countries BMC Med Ethics 2010 11 4 12 10.1186/1472-6939-11-4 20331891 PMC2859353

[B18] Slaughter LM The Genetic Information Nondiscrimination Act: Why your Personal Genetics are Still Vulnerable to Discrimination Surg Clin N Am 2008 88 723 738 10.1016/j.suc.2008.04.004 18672138

[B19] Wade CH Kalfoglou AL When Do Genetic Researchers Have a Duty to Re-contact Study Participants? Am J Bioeth 2006 6 26 27 17085400 10.1080/15265160600935746

[B20] Chin L Gray JW Translating insights from the cancer genome into clinical practice Nature 2008 452 553 563 10.1038/nature06914 18385729 PMC2730524

[B21] Guttmacher AE McGuire AL Ponder B Stefansson K Personalized genomic information: preparing for the future of genetic medicine Nat Rev Genet 2010 11 161 165 20065954 10.1038/nrg2735

[B22] Murphy J Scott J Kaufman D Geller G Hudson K Public Expectations for Return of Results from Large-Cohort Genetic Research Am J Bioeth 2008 8 36 43 19061108 10.1080/15265160802513093 PMC2682364

[B23] Applebaum PS Roth LH Lidz CW Benson P Winslade W False hopes and best data: consent to research and the therapeutic misconception Hasting Center Rep 1987 17 20 24 3294743

[B24] Ravitsky V Wilfond BS Disclosing Individual Genetic Results to Research Participants Am J Bioeth 2006 6 8 17 17085395 10.1080/15265160600934772

[B25] Bovenberg JA Meulenkamo T Smets EM Gevers JKM Your Biobank, Your Doctor? The Right to full disclosure of population biobank findings Genomics, Society and Policy 2009 5 55 79

[B26] Miller FG Mello MM Joffe S Incidental findings in human subjects research: What do investigators owe research participants? J Law Med Ethics 2008 36 271 10.1111/j.1748-720X.2008.00269.x 18547194 PMC2610459

[B27] Knoppers BM Joly Y Simard J Durocher F The emergence of an ethical duty to disclose genetic research results: international perspectives Eur J Hum Genet 2006 13 1170 1178 10.1038/sj.ejhg.5201690 16868560

[B28] McGuire AL Lupski JR Personal genome research: what should the participant be told? Trends Genet 2010 26 199 201 10.1016/j.tig.2009.12.007 20381895 PMC2868334

[B29] Bredenoord AL Kroes HY Cuppen E Parker M van Delden JJM Disclosure of individual genetic data to research participants: the debate reconsidered Trends Genet 2011 27 41 7 10.1016/j.tig.2010.11.004 21190750

[B30] United Nations Educational,Scientific and Cultural Organization International Declaration on Human Genetic Data 2003 http://www.unesco.org/new/en/unesco human-sciences/themes/bioethics/human-genetic-data/

[B31] World Medical Association Declaration of Helsinki, Ethical Principles for Medical Research involving Human Subjects, as amended by the 59th WMA General Assembly in Seoul 2008 http://www.wma.net/en/30publications/10policies/b3/index.html

[B32] Robison K Application of second-generation sequencing to cancer genomics Brief Bioinform 2010 11 524 534 10.1093/bib/bbq013 20427421

[B33] Ciruli ET Goldstein DB Uncovering the roles of rare variants in common disease through whole-genome sequencing Nat Rev Genet 2010 11 415 425 10.1038/nrg2779 20479773

