# Supplementary material for: Qualitative thematic analysis of consent forms used in cancer genome sequencing
Source: BMC Med Ethics. 2011 Jul 19;12:14. doi: 10.1186/1472-6939-12-14 (PMC3170865; doi:10.1186/1472-6939-12-14)
Supplement: Additional file 1 — Summary of published cancer genome sequencing projects. Provides information on notable cancer genome sequencing projects that have been published. [file 1472-6939-12-14-S1.PDF]

Additional File 1: Summary of published cancer genome sequencing projects

	Type	Source	Target	Highlights of Genetic Findings	Ref
Myeloid neoplasms	Acute myeloid leukemia (AML) - M1	Primary tumour	Genome	First cancer genome sequenced by massively parallel sequencing. Identified mutations in *FLT3* and *NPM1,* previously known drivers of tumorigenesis.	[1]
		Primary tumour	Genome	*IDH1* mutations were present in 13 of 80 (16%) of cytologically normal AML genomes.	[2]
		Primary tumour	Genome	Newer sequencing techniques identified a base pair insertion in DNA methyltransferase 3A (*DNMT3A)* not found in the original AML-M1 genome first published by Ley et al. in 2008. *DNMT3A* mutations are present in 62 of 281 (22.1%) AMLs and are independently associated with poor outcome.	[3]
	Acute myeloid leukemia (AML) - M5	9 primary tumours	Exome	Identified a recurrent somatic mutation affecting the *DNMT3A* in 23 of 112 (20.5%) AML-M5 tumors. Further analysis of *DNMT3A* in AML-M4 cases revealed mutations in 9 of 66 (13.6%). Mutations in *DNMT3A* were shown to affect the protein's enzymatic activity and alter expression profiles. Leukemias with *DNMT3A* are associated with poor prognosis.	[4]
Lymphoid neoplasms	Follicular lymphoma (FL) and germinal-center B-cell type diffuse large B-cell lymphoma (GBC DLBCL)	1 primary FL 31 primary GCB DLBCLs and 7 GCB DLBCL cell lines	Exome and transcriptome	Identified a recurrent somatic mutation affecting the polycomb-group oncogene *EZH2,* encoding a histone methyltransferase, in 18 of 83 (21.7%) DLBCLs and 16 of 221 (7.2%) FLs.	[5]
	Activated B-cell like diffuse large B-cell lymphoma (ABC DLBCL)	4 primary tumours	Transcriptome	Identified a single mutation, L265P, in *MYD88* that was present in 111 of 382 lymphomas (29%). Additionally L265P was present in 9% of mucosa-associated lymphod tissue lymphomas. Analysis of this mutation demonstrated a gain-of-function promoting cell survival through the NF-kB and JAK-STAT3 pathway.	[6]
	Multiple myeloma (MM)	38 primary tumours	Genome and exome	Analysis of 23 multiple myeloma genomes as well as 16 multiple myeloma exomes (including one that was analysed by both methods) revealed mutations involving genes that regulate: RNA processing, protein homeostasis, the NF-kB pathway, gene imprinting and the coagulation cascade. Additionally, mutations in *BRAF* and in non-coding regions were discovered.	[7]
	Chronic lymphocytic leukemia (CLL)	4 primary tumours	Genome	Identified 45 genes with mutations in protein-coding sequences. Analysis of these 45 genes in an additional 363 CLL patients identified 4 genes that are recurrently mutated: *NOTCH1, MYD88, XPO1 and KLHL6*. Mutations identified in *NOTCH1* and *MYD88* are activating mutations present in 31/255 (12.2%) and 9/310 (2.9%) respectively. Mutations in *NOTCH1* are associated with decreased overall survival.	[8]
	Hairy-cell leukemia	Primary tumor	Exome	Exome sequencing identified 5 nonsynonymous mutations one of which	[9]

	(HCL)			was *BRAF* V600E. An additional 47 patients with HCL were examined. All had *BRAF* V600E mutations and virtually all were present in the original clone. None were found in 195 patients with other peripheral B-cell lymphomas and leukemias.	
	Classical Hodgkin lymphoma	2 cancer cell lines	Transcriptome	Identified a gene fusion involving *CIITA* that was found to be recurrent in 29 of 77 B-cell lymphomas (38%) and 8 of 55 (15%) classical Hodgkin lymphomas. *CIITA* gene fusions result in down-regulation of surface HLA class II expression and up-regulation of ligands of PDL1/2 allowing the tumour cell to escape immune detection.	[10]
Breast	Lobular breast cancer	Lung metastasis	Genome and transcriptome	Five mutations (in *ABCB11, HAUS3, SLC24A4, SNX4* and *PALB2*) were prevalent in both the primary and the metastatic tumour. RNA-editing events contribute to the transcriptional variation of lobular breast cancer.	[11]
	Basal-like breast cancer	Primary tumour, brain metastasis and mouse xenograft	Genome	Point mutations were predominantly C>T/G>A transitions. Comparing the mutational spectrum of the primary tumour with that of the metastases and xenograft, suggested that all the mutations necessary for metastases were already present in the primary tumour.	[12]
	Triple negative breast cancer	2 primary tumours and liver metastasis	Copy number variation of a single-nucleus for 100 cells of each tumour	Phylogenetic reconstruction demonstrated that tumour progression is characterized by a "punctuated clonal evolution". Findings also suggest that metastatic potential is achieved in the late stages of tumour evolution.	[13]
Ovarian	Granuolsa-cell tumour (GCT)	4 primary tumours	Transcriptome	Identified a single recurrent mutation 402C>G (C134W) in *FOXL2* in all 4 GCTs. Sequencing of an additional 89 GCTs revealed the same mutation in 86 (97%).	[14]
	Ovarian clear-cell carcinoma (OCCC)	8 primary tumours	Exome	Identified *PPP2R1A* and *ARID1A* mutations in two tumours. Resequencing 42 OCCCs revealed *PPP2R1A* mutations were present in 7% and *ARID1A* mutations in 57% of OCCCs.	[15]
		18 OCCCs and 1 OCCC cell line	Exome and transcriptome	*ARID1A* mutations were present in 55 of 119 (46%) ovarian clear-cell carcinomas, and 10 of 33 (30%) endometrioid carcinomas.	[16]
	High-grade serous ovarian cancer	316 HGS-OvCa	Exome, genomic rearrangements, transcriptome, methylome	HGS-Ovarian cancers are characterized by mutations in TP53 (96% of cases) and mutations in genes involved in DNA repair by homologous recombination (50%). These finding may explain the high prevalence of chromosomal rearrangements in this cancer. 20% of cases either had mutated or hypermethylated BRCA1/2 but not both. Those with mutated BRCA1/2 had significantly better overall survival compared to those with the wild-type allele. Whereas those with hypermethylated BRCA1/2 had similar survival rates compared to wild-type.	[17]

Additional File 1: Summary of published cancer genome sequencing projects

Lung	Small-cell lung cancer	NCI-H209 cell line	Genome	Tabocco carcinogens predominantly cause G>T/C>A transversions, preferentially at methylated CpGs. Mutational signatures showed evidence of transcription-coupled nucleotide excision repair as well as expression-coupled repair.	[18]
	Non-small-cell lung cancer	Primary tumour	Genome	The mutational signature was similar to that observed in small-cell lung cancer. The predominant point mutations were G>T/C>A transversions and in methylated CpGs with evidence of transcription-coupled repair.	[19]
	Malignant pleural mesothelioma	Primary tumour	Genome	A *DPP10* deletion was identified in the primary tumour. Resequencing of this gene in 53 tumour samples was detected in 31 (55%) and showed loss of *DPP10* is associated with a poorer survival.	[20]
Melanoma	Melanoma	COLO-829 cell line, a malignant cell line	Genome	The mutational imprint left by UV damage is characterized predominantly by C>T/G>A transitions, particularly between two adjacent pyrimidines. These mutations occur more frequently at the 3' base of a pyrimidine dinucleotide and in CpG dinucleotides. There was also evidence for transcription-coupled nucleotide excision repair.	[21]
	Uveal Melanoma class 2	2 tumours with monosomy 3	Exome	Both tumours contained inactivating mutations in BRCA1-associated protein 1 (*BAP1*) at 3p21.1. An additional 26 of 31 (84%) class 2 (high metastatic risk) UMs showed mutations in BAP1, while only 1 of 26 class 1 (low metastatic risk) UMs contained BAP1 mutations. Additionally, one patient had a germline *BAP1* mutation, suggesting that germline mutations in *BAP1* could be a novel cancer predisposition gene. *BAP1* regulates a number of genes involved in metastatic transformation.	[22]
Brain	Glioblastoma multiforme	21 primary tumours	Exome and transcriptome	Mutations in *IDH1* were found in 18 of 149 (12%) GBMs and all affect amino acid R132. Mutations affecting R132 are associated with a better prognosis.	[23]
	Medulloblastoma (MB)	17 primary tumours,<br>4 xenografts,<br>1 cell line<br>7 metastic MBs	Exome	The number of nonsilent (nonsynonymous, missense, nonsense, indels or splice site) mutations per medulloblastoma, a childhood cancer, was only 8.3, five to ten times fewer mutations compared to adult solid tumours. The discovery of recurring mutations in *MLL2* and *MLL3*, genes regulating transcription and chromatin remodeling, suggests that MB is caused by mechanisms that subvert normal brain development, explaining its rarity in adult populations.	[24]
Renal	Clear cell renal carcinoma (ccRCC)	7 primary tumours	Exome	Identified truncating mutations in *PBRM1.* Sequencing of an additional 257 renal cell carcinomas identified truncating mutations in 88 (34%). *PBRM1* maps to 3p, the same position as tumour suppressor genes, *VHL* and *SETD2*. This may be why 3p loss-of-heterozygosity is commonly seen in ccRCC.	[25]
Gastro-intestinal	Colorectal cancer	3 cell lines and 8 xenografts all derived from	Exome	*IDH1* mutations affecting R132 were overlooked as driver mutations, however they were retrospectively recognized after discovering similar mutations in glioblastoma multiforme (Parsons 2008)	[26]

		liver metastases			
	Pancreatic adenocarcinoma	24 primary tumours	Exome	Grouped mutated genes into 69 gene sets of which 31 could be further grouped into 12 core signalling pathways. Expression data of these 31 gene sets suggest that they contribute to pancreatic tumourigenesis.	[27]
		24 primary tumours	Exome	Identified one pancreatic tumor, which harbored both a germline and somatically acquired deleterious mutation in *PALB2*. Sequencing *PALB2* in 96 patients with familial pancreatic cancer identified 3 patients with germline *PALB2* truncating mutations, while none were found in 1084 controls.	[28]
		Primary tumour and metastasis from thirteen patients	Genomic rearrangements by paired-end sequencing	"Fold-back inversions" are a distinct pattern of genomic instability in pancreatic cancer indicative of telomere erosion and dysregulation of the G1-to-S transition.	[29]
		Primary tumour and metastases from seven patients	Genome	Analysis of the genome of pancreatic cancers at various spatial and temporal stages of its evolution demonstrate that it is initiated by a single parental clone, from which progressor mutations accumulate and drive clonal evolution. Estimated time from the initiated tumour cell to parental clone is 11.7 years, while another 3.4 years elapses before subclones with metastatic potential are achieved. This suggests that metastatic cells arise late in tumour development.	[30]
	Pancreatic neuroendocrine tumour (PanNET)	10 sporadic PanNETs	Exome	Identified and validated mutations in 58 PanNETs in chromatin remodeling genes (*MEN1 in 44% and either DAXX* or *ATRX in 43%)* as well as mutations involving the mTOR pathway (14%).	[31]
	Hepatocellular carcinoma (HCC)	1 primary tumour	Genome and exome	Whole genome sequencing of a hepatitis C virus induced HCC revealed a predominance of T>C/A>G transitions with evidence of transcription-coupled repair. Exome sequencing of the same tumour sample at a higher sequence depth revealed sub-clones with mutations in the tumour suppressor gene, *TSC1.*	[32]
Prostate	Prostate	7 tumours	Genome	Complex genomic rearrangements in prostate cancer are characterized by balanced translocations. Furthermore, a single closed chain of rearrangements can disrupt multiple known cancer genes.	[33]

Additional File 1: Summary of published cancer genome sequencing projects

References

1. Ley TJ, Mardis ER, Ding L, Fulton B, McLellan MD, Chen K, Dooling D, Dunford-Shore BH, McGrath S, Hickenbotham M *et al*: **DNA sequencing of a cytogenetically normal acute myeloid leukaemia genome**. *Nature* 2008, **456**(7218):66-72.
2. Mardis ER, Ding L, Dooling DJ, Larson DE, McLellan MD, Chen K, Koboldt DC, Fulton RS, Delehaunty KD, McGrath SD *et al*: **Recurring mutations found by sequencing an acute myeloid leukemia genome**. *N Engl J Med* 2009, **361**(11):1058-1066.
3. Ley TJ, Ding L, Walter MJ, McLellan MD, Lamprecht T, Larson DE, Kandoth C, Payton JE, Baty J, Welch J *et al*: **DNMT3A mutations in acute myeloid leukemia**. *N Engl J Med* 2010, **363**(25):2424-2433.
4. Yan XJ, Xu J, Gu ZH, Pan CM, Lu G, Shen Y, Shi JY, Zhu YM, Tang L, Zhang XW *et al*: **Exome sequencing identifies somatic mutations of DNA methyltransferase gene DNMT3A in acute monocytic leukemia**. *Nat Genet* 2011, **43**(4):309-315.
5. Morin RD, Johnson NA, Severson TM, Mungall AJ, An J, Goya R, Paul JE, Boyle M, Woolcock BW, Kuchenbauer F *et al*: **Somatic mutations altering EZH2 (Tyr641) in follicular and diffuse large B-cell lymphomas of germinal-center origin**. *Nat Genet* 2010, **42**(2):181-185.
6. Ngo VN, Young RM, Schmitz R, Jhavar S, Xiao W, Lim KH, Kohlhammer H, Xu W, Yang Y, Zhao H *et al*: **Oncogenically active MYD88 mutations in human lymphoma**. *Nature* 2011, **470**(7332):115-119.
7. Chapman MA, Lawrence MS, Keats JJ, Cibulskis K, Sougnez C, Schinzel AC, Harview CL, Brunet JP, Ahmann GJ, Adli M *et al*: **Initial genome sequencing and analysis of multiple myeloma**. *Nature* 2011, **471**(7339):467-472.
8. Puente XS, Pinyol M, Quesada V, Conde L, Ordonez GR, Villamor N, Escaramis G, Jares P, Bea S, Gonzalez-Diaz M *et al*: **Whole-genome sequencing identifies recurrent mutations in chronic lymphocytic leukaemia**. *Nature* 2011.
9. Tiacci E, Trifonov V, Schiavoni G, Holmes A, Kern W, Martelli MP, Pucciarini A, Bigerna B, Pacini R, Wells VA *et al*: **BRAF mutations in hairy-cell leukemia**. *N Engl J Med* 2011, **364**(24):2305-2315.
10. Steidl C, Shah SP, Woolcock BW, Rui L, Kawahara M, Farinha P, Johnson NA, Zhao Y, Telenius A, Neriah SB *et al*: **MHC class II transactivator CIITA is a recurrent gene fusion partner in lymphoid cancers**. *Nature* 2011, **471**(7338):377-381.
11. Shah SP, Morin RD, Khattra J, Prentice L, Pugh T, Burleigh A, Delaney A, Gelmon K, Guliany R, Senz J *et al*: **Mutational evolution in a lobular breast tumour profiled at single nucleotide resolution**. *Nature* 2009, **461**(7265):809-813.
12. Ding L, Ellis MJ, Li S, Larson DE, Chen K, Wallis JW, Harris CC, McLellan MD, Fulton RS, Fulton LL *et al*: **Genome remodelling in a basal-like breast cancer metastasis and xenograft**. *Nature* 2010, **464**(7291):999-1005.
13. Navin N, Kendall J, Troge J, Andrews P, Rodgers L, McIndoo J, Cook K, Stepansky A, Levy D, Esposito D *et al*: **Tumour evolution inferred by single-cell sequencing**. *Nature* 2011, **472**(7341):90-94.
14. Shah SP, Kobel M, Senz J, Morin RD, Clarke BA, Wiegand KC, Leung G, Zayed A, Mehl E, Kalloger SE *et al*: **Mutation of FOXL2 in granulosa-cell tumors of the ovary**. *N Engl J Med* 2009, **360**(26):2719-2729.
15. Jones S, Wang TL, Shih Ie M, Mao TL, Nakayama K, Roden R, Glas R, Slamon D, Diaz LA, Jr., Vogelstein B *et al*: **Frequent mutations of chromatin remodeling gene ARID1A in ovarian clear cell carcinoma**. *Science* 2010, **330**(6001):228-231.
16. Wiegand KC, Shah SP, Al-Agha OM, Zhao Y, Tse K, Zeng T, Senz J, McConechy MK, Anglesio MS, Kalloger SE *et al*: **ARID1A mutations in endometriosis-associated ovarian carcinomas**. *N Engl J Med* 2010, **363**(16):1532-1543.
17. **Integrated genomic analyses of ovarian carcinoma**. *Nature* 2011, **474**(7353):609-615.
18. Pleasance ED, Stephens PJ, O'Meara S, McBride DJ, Meynert A, Jones D, Lin ML, Beare D, Lau KW, Greenman C *et al*: **A small-cell lung cancer genome with complex signatures of tobacco exposure**. *Nature* 2010, **463**(7278):184-190.
19. Lee W, Jiang Z, Liu J, Haverty PM, Guan Y, Stinson J, Yue P, Zhang Y, Pant KP, Bhatt D *et al*: **The mutation spectrum revealed by paired genome sequences from a lung cancer patient**. *Nature* 2010, **465**(7297):473-477.
20. Bueno R, De Rienzo A, Dong L, Gordon GJ, Hercus CF, Richards WG, Jensen RV, Anwar A, Maulik G, Chirieac LR *et al*: **Second generation sequencing of the mesothelioma tumor genome**. *PLoS One* 2010, **5**(5):e10612.
21. Pleasance ED, Cheetham RK, Stephens PJ, McBride DJ, Humphray SJ, Greenman CD, Varela I, Lin ML, Ordonez GR, Bignell GR *et al*: **A comprehensive catalogue of somatic mutations from a human cancer genome**. *Nature* 2010, **463**(7278):191-196.
22. Harbour JW, Onken MD, Roberson ED, Duan S, Cao L, Worley LA, Council ML, Matatall KA, Helms C, Bowcock AM: **Frequent mutation of BAP1 in metastasizing uveal melanomas**. *Science* 2010, **330**(6009):1410-1413.
23. Parsons DW, Jones S, Zhang X, Lin JC, Leary RJ, Angenendt P, Mankoo P, Carter H, Siu IM, Gallia GL *et al*: **An integrated genomic analysis of human glioblastoma multiforme**. *Science* 2008, **321**(5897):1807-1812.
24. Parsons DW, Li M, Zhang X, Jones S, Leary RJ, Lin JC, Boca SM, Carter H, Samayoa J, Bettegowda C *et al*: **The genetic landscape of the childhood cancer medulloblastoma**. *Science* 2011, **331**(6016):435-439.

25. Varela I, Tarpey P, Raine K, Huang D, Ong CK, Stephens P, Davies H, Jones D, Lin ML, Teague J *et al*: **Exome sequencing identifies frequent mutation of the SWI/SNF complex gene PBRM1 in renal carcinoma**. *Nature* 2011, **469**(7331):539-542.
26. Sjoblom T, Jones S, Wood LD, Parsons DW, Lin J, Barber TD, Mandelker D, Leary RJ, Ptak J, Silliman N *et al*: **The consensus coding sequences of human breast and colorectal cancers**. *Science* 2006, **314**(5797):268-274.
27. Jones S, Zhang X, Parsons DW, Lin JC, Leary RJ, Angenendt P, Mankoo P, Carter H, Kamiyama H, Jimeno A *et al*: **Core signaling pathways in human pancreatic cancers revealed by global genomic analyses**. *Science* 2008, **321**(5897):1801-1806.
28. Jones S, Hruban RH, Kamiyama M, Borges M, Zhang X, Parsons DW, Lin JC, Palmisano E, Brune K, Jaffee EM *et al*: **Exomic sequencing identifies PALB2 as a pancreatic cancer susceptibility gene**. *Science* 2009, **324**(5924):217.
29. Campbell PJ, Yachida S, Mudie LJ, Stephens PJ, Pleasance ED, Stebbings LA, Morsberger LA, Latimer C, McLaren S, Lin ML *et al*: **The patterns and dynamics of genomic instability in metastatic pancreatic cancer**. *Nature* 2010, **467**(7319):1109-1113.
30. Yachida S, Jones S, Bozic I, Antal T, Leary R, Fu B, Kamiyama M, Hruban RH, Eshleman JR, Nowak MA *et al*: **Distant metastasis occurs late during the genetic evolution of pancreatic cancer**. *Nature* 2010, **467**(7319):1114-1117.
31. Jiao Y, Shi C, Edil BH, de Wilde RF, Klimstra DS, Maitra A, Schulick RD, Tang LH, Wolfgang CL, Choti MA *et al*: **DAXX/ATRX, MEN1, and mTOR pathway genes are frequently altered in pancreatic neuroendocrine tumors**. *Science* 2011, **331**(6021):1199-1203.
32. Totoki Y, Tatsuno K, Yamamoto S, Arai Y, Hosoda F, Ishikawa S, Tsutsumi S, Sonoda K, Totsuka H, Shirakihara T *et al*: **High-resolution characterization of a hepatocellular carcinoma genome**. *Nat Genet* 2011, **43**(5):464-469.
33. Berger MF, Lawrence MS, Demichelis F, Drier Y, Cibulskis K, Sivachenko AY, Sboner A, Esgueva R, Pflueger D, Sougnez C *et al*: **The genomic complexity of primary human prostate cancer**. *Nature* 2011, **470**(7333):214-220.
